# Effectiveness of Gelatin-Thrombin Matrix Sealants (Floseal®) on Postoperative Spinal Epidural Hematoma during Single-Level Lumbar Decompression Using Biportal Endoscopic Spine Surgery: Clinical and Magnetic Resonance Image Study

**DOI:** 10.1155/2020/4801641

**Published:** 2020-07-08

**Authors:** Ju-Eun Kim, Hyun-Seung Yoo, Dae-Jung Choi, Eugene J. Park, Jin-Ho Hwang, Jeong-Duk Suh, Jun-Hyug Yoo

**Affiliations:** ^1^Department of Orthopedic Surgery, Himnaera Hospital, Busan, Republic of Korea; ^2^Department of Orthopedic Surgery, Daegu Fatima Hospital, Daegu, Republic of Korea; ^3^Department of Orthopedic Surgery, Kyungpook National University hospital, Daegu, Republic of Korea

## Abstract

**Background:**

Symptomatic postoperative spinal epidural hematoma (PSEH) is a devastating complication that could develop after lumbar decompression surgery. PSEH can also develop after biportal endoscopic spine surgery (BESS), one of the recently introduced minimally invasive spine surgery techniques. Gelatin-thrombin matrix sealant (GTMS) is commonly used to prevent PSEH. This study aimed at analyzing the clinical and radiological effects of GTMS use during BESS.

**Methods:**

A total of 206 patients with spinal stenosis who underwent decompression by BESS through a posterior interlaminar approach from October 2015 to September 2018 were enrolled in this study. Postoperative magnetic resonance imaging (MRI) was performed in all patients for evaluation of PSEH. Patients in whom GTMS was not used during surgery were assigned to Group A, and those in whom GTMS was used were classified as Group B. In the clinical evaluation, the visual analog scale (VAS) of the leg and back, Oswestry Disability Index (ODI), and modified MacNab criteria were used. The incidence rate and degree of dural compression of PSEH on postoperative MRI were measured.

**Results:**

The average age of the patients was 68.1 ± 11.2 (42–89) years. The overall incidence rate of PSEH was 20.9% (43/206). The incidence rates in Groups A and B were 26.4% and 13.6%, respectively, showing a significant difference (*p* = 0.023). The VAS-leg and ODI improvement was significantly different depending on the intraoperative use of GTMS. However, there was no statistically significant difference between the two groups in terms of the VAS-back improvement. Groups A and B showed “good” and “excellent” rates according to the modified MacNab criteria in 79.4% and 87.6% of patients, respectively, showing statistically significant difference (*p* = 0.049). In Group A, two patients underwent revision surgery due to PSEH, while none in Group B had such event.

**Conclusion:**

Intraoperative use of GTMS during BESS may be related to reduction in the occurrence rate of PSEH. Specifically, patients with GTMS appliance showed marked decrease in the occurrence of PSEH and had better clinical outcomes.

## 1. Introduction

Postoperative spinal epidural hematoma (PSEH) is one of the most common early complications of spine surgery that can cause neurological symptoms [1, 2]. The incidence of PSEH is not extremely high; however, it can cause severe neurological deficits and can lead to cases that may require prompt treatment [3]. The epidural venous plexus is thinner in patients with chronic lumbar spinal stenosis than in normal patients; therefore, patients with spinal stenosis have increased risk of bleeding due to rupture of the venous plexus during decompression [4]. Inadequate hemostasis can cause severe PSEH, which may result in complications, such as cauda equina syndrome, leading to a more unsatisfactory clinical outcome.

Fibrosis that forms around the dura is considered one of the critical factors of failed back syndrome and arises from the overproduction of postoperative scar tissue [5]. Excessive epidural fibrosis can develop after laminectomy due to insufficient hemostasis; thus, meticulous hemostasis is an essential step. A gelatin-thrombin matrix sealant (GTMS) is a well-known biocompatible hemostatic matrix composed of bovine gelatin matrix and human-derived thrombin used during spine surgery [6]. Such GTMSs are known to promote fibrin formation and coagulation [7]. This agent acts on two areas in the coagulation cascade: contact activation and thrombin activation.

Recently, the use of an endoscope has gained interest in the field of minimally invasive surgery. For the treatment of spinal stenosis, biportal endoscopic spine surgery (BESS) was introduced as an alternative method for microscopic unilateral laminotomy for bilateral decompression (ULBD) [8–13]. The basic concept of the BESS is similar to those of arthroscopic and laparoscopic surgeries, in which an endoscope is inserted through a viewing portal, and surgical instruments are inserted into a separate working portal. The advantage of BESS is that the early postoperative clinical outcome is more favorable than that in conventional open surgery due to less paraspinal muscle dissection and retraction [8]. Although BESS is performed with minimal manipulation of the soft tissues, it is reported that radiological PSEH develops at a rate of 24.7%. Since patients with PSEH show poorer postoperative clinical outcomes, prevention of PSEH is essential [14, 15]. Compared to discectomy, in procedures that require more manipulation of the bone and soft tissue, such as in bilateral decompression and transforaminal lumbar interbody fusion, the incidence of PSEH is reported to be higher [14]. Based on previous studies on adequate hemostasis using GTMS [7], this study aimed at evaluating the effect of GTMS use during BESS on the incidence of PSEH.

## 2. Materials and Methods

The study was conducted after obtaining approval from the Institutional Review Board. All patients who underwent BESS provided informed consent to undergo the surgery. Electronic medical records and preoperative and postoperative magnetic resonance imaging (MRI) findings were retrospectively obtained and analyzed by board-certified orthopedic surgeons. Among the patients who underwent BESS, 206 consecutive patients who underwent single-level ULBD with BESS technique through the posterior interlaminar approach from October 2015 to September 2018 were enrolled. The inclusion criteria for surgery were as follows: (1) central or lateral recess stenosis without foraminal stenosis; (2) preoperative symptoms of lower extremity radiating pain or neurogenic claudication; and (3) refractory to conservative treatment including use of medications, such as analgesics, nonsteroidal anti-inflammatory drugs, and gabapentinoids, or epidural injections for at least 3 months. The exclusion criteria were as follows: (1) lumbar foraminal stenosis treated with foraminal decompression; (2) spondylolytic spondylolisthesis or instability of >5° of angulation or >3 mm of translation on dynamic radiography; (3) infection or fracture; (4) complications other than PSEH, such as dural tear; and (5) <1 year of follow-up.

**Figure 1:**
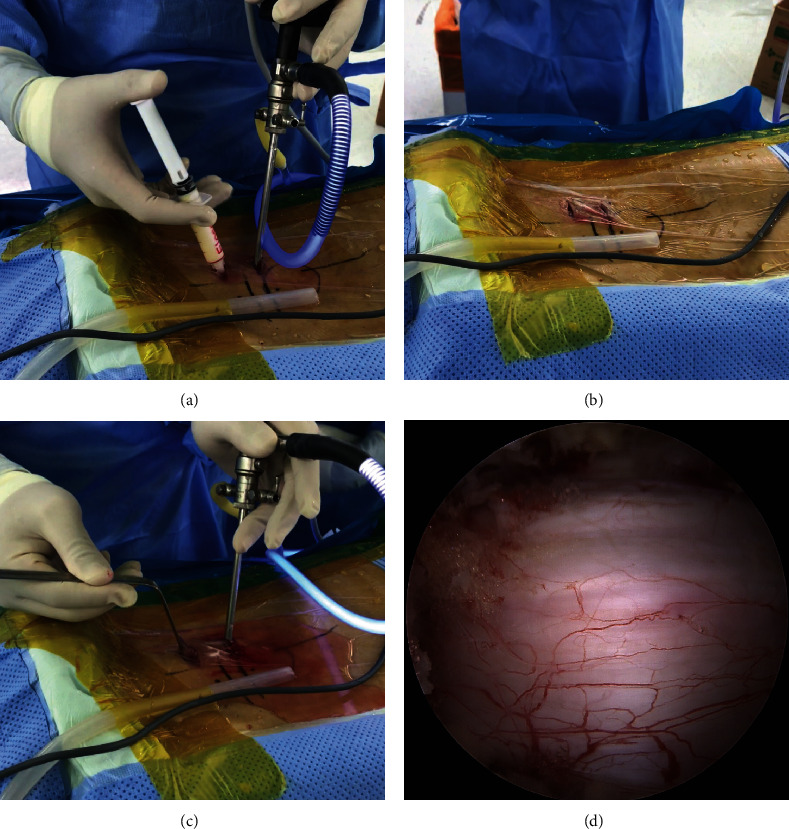
GTMS usage during BESS. (a) GTMS injected into the spinal canal after stopping water infusion. (b) Wait 3 minutes after injection of the GTMS and removal of the endoscope. (c) GTMS and hematoma were washed out using continuous saline irrigation. (d) Clearance of GTMS from the dura confirmed on the intraoperative photograph. (GTMS: gelatin-thrombin matrix sealant, BESS: biportal endoscopic spine surgery).

**Figure 2:**
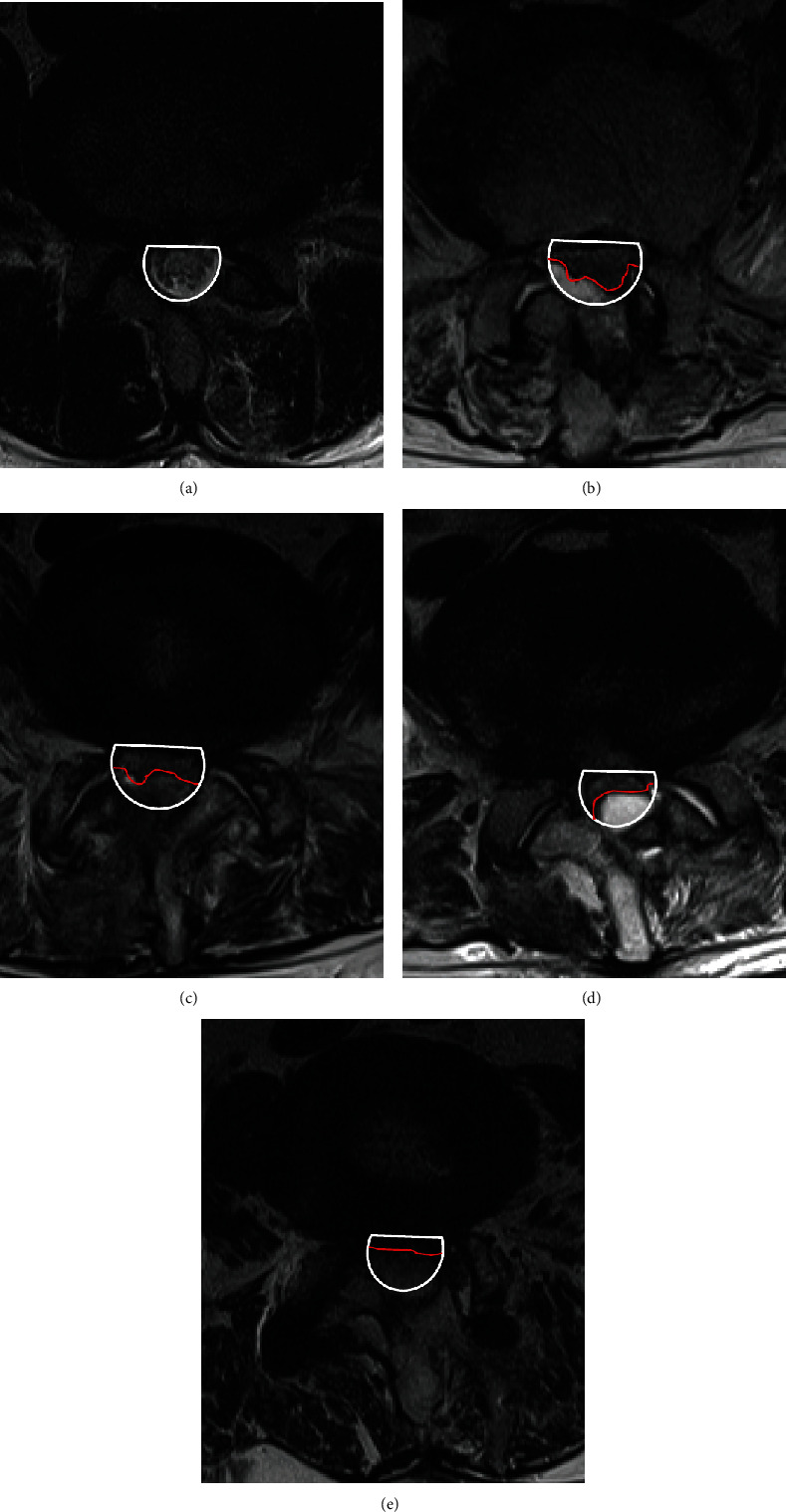
Measuring the severity of PSEH on T2-weighted axial MRI. (a) Grade 0, (b) Grade I, (c) Grade II, (d) Grade III, and (e) Grade IV. (PSEH: postoperative spinal epidural hematoma, MRI: magnetic resonance image).

During the initial period of performing BESS, we did not routinely use Floseal® (Baxter International, Inc., Deerfield, IL, USA), since adequate hemostasis was achieved intraoperatively. However, since postoperative MRI of these patients showed minor epidural hematoma, in some cases with neurologic symptoms, we considered using GTMS to reduce the incidence of epidural hematoma since May 2017. Patients in whom GTMS was not used during surgery were assigned to Group A, and those in whom GTMS was used were classified as Group B. In Group B, 5 mL of GTMS was injected above the dura through the working portal after stopping the water infusion by locking the valve on the endoscope. Three minutes after injection, by saline infusion, the complex of GTMS and blood clots were drained through a semitubular retractor placed in the working portal (Figure 1) (Vid. 1). The granular material that was not incorporated in the blood clot was carefully removed by irrigation and probing, reducing the compression of the dural sac by the GTMS. The drain was removed 1 or 2 days after surgery when the daily amount was <50 mL. Postoperative MRI was routinely performed on the day of drain removal using 1.5 T MRI unit (Avanto®, Siemens, Munich, Germany). The MRI had a slice thickness of 4 mm in both the sagittal and axial images, and T1-weighted sagittal and axial images and T2-weighted sagittal and fat-suppressed axial images were obtained. T2 axial images on postoperative MRI were used to measure the dimension of the maximal dural compression due to hematoma, performed by an experienced radiologist. While no follow-up MRI was routinely performed besides that on the day of drain removal, additional follow-up MRI was performed in patients who underwent revision surgery for symptomatic PSEH after revision surgery. PSEH was defined as hematoma compressing the dural sac in MRI T2-weighted axial images and was classified as Grade 0 (no hematoma), Grade I (<25% compression of the spinal canal), Grade II (25–50% compression of the spinal canal), Grade III (50–75% compression of the spinal canal), and Grade IV (>75% compression of the spinal canal) [15](Figure 2). Utilizing postoperative MRI, the occurrence rate and grade of PSEH in Groups A and B were comparatively analyzed by the senior author blinded to the use of GTMS. Moreover, the visual analog scale (VAS) of the leg and back, Oswestry Disability Index (ODI), and modified MacNab criteria were used to measure the clinical outcome preoperatively, 3 months postoperatively, and at the final follow-up of at least 1 year postoperatively. All statistical analyses were performed using SPSS version 22 (IBM Corp., Armonk, New York, USA). Values are presented as mean ± standard deviation. The differences in radiological and clinical outcomes between Groups A and B were analyzed using a paired *t*-test and repeated-measures analysis of variance.

## 3. Results

**Figure 3:**
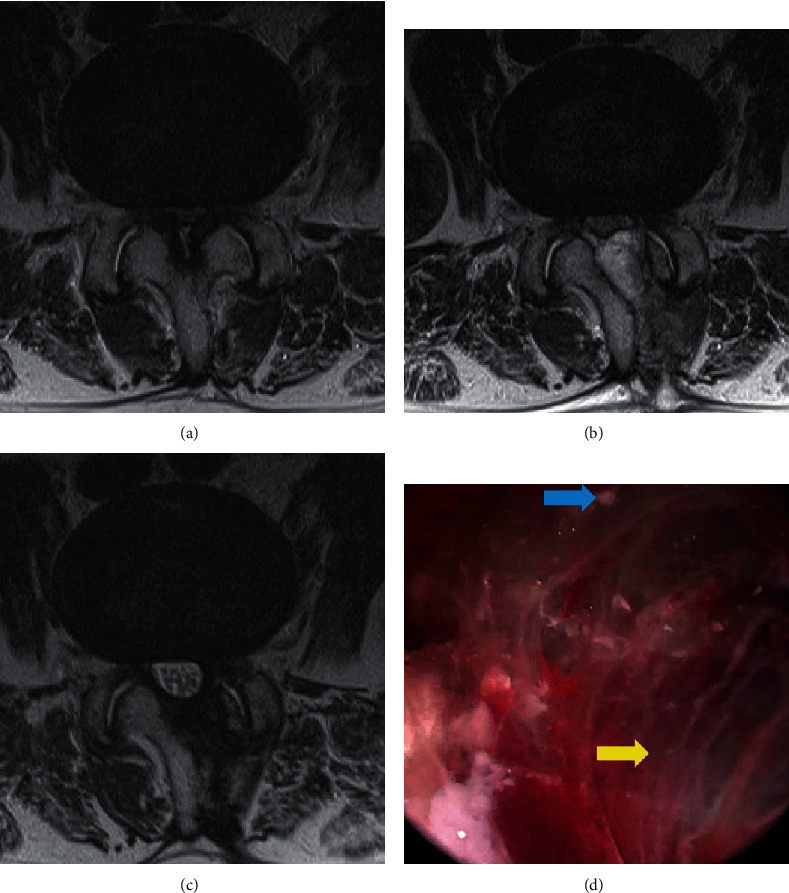
A patient who underwent BESS decompression for L4–5 spinal stenosis and additional revision surgery (hematoma evacuation) due to aggravated neurological symptoms caused by PSEH. (a) Preoperative T2-weighted magnetic resonance imaging showing central stenosis. (b) Postoperative T2-weighted MRI showing grade III PSEH. (c) Follow-up MRI 6 months after revision surgery showing removed PSEHs. (d) Intraoperative image during revision surgery. Yellow arrow: PSEH. Blue arrow: bone debris. (BESS: biportal endoscopic spine surgery, PSEH: postoperative spinal epidural hematoma, MRI: magnetic resonance image).

**Figure 4:**
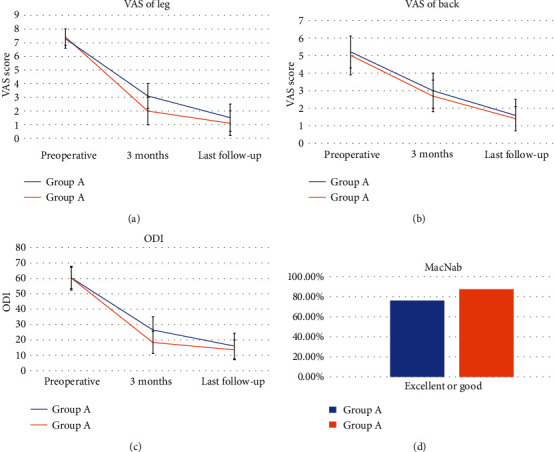
Clinical outcome improvement. (a) VAS of the leg. (b) VAS score of back. (c) ODI (%). (d) Modified MacNab criteria. (VAS: visual analog scale, ODI: Oswestry Disability Index).

A total of 206 patients were enrolled in the study, of which 107 (51.9%) were female and 99 (48.1%) were male. The average age was 68.1 ± 11.1 (49–89) years. The index level of surgery was L2–3 in 5 patients (2.4%), L3–4 in 28 patients (13.6%), L4–5 in 123 patients (78.6%), and L5–S1 in 11 patients (5.3%). Overall, PSEH was present in 43 patients (20.9%). Of the 43 patients, 19 (9.2%) had Grade I, 19 (9.2%) had Grade II, and 5 (2.4%) had Grade III (Table 1). Two patients with Grade III PSEH had postoperative neurologic symptoms, including radiating pain on both lower extremities that were intractable to conservative treatment and thus underwent revision surgery of PSEH evacuation. The two patients who underwent revision surgery both showed Grade III PSEH. Hematoma evacuation revision surgery was performed through the previous portal used in the BESS technique (Figure 3). Although revision surgeries were performed in patients with PSEH in Groups A (2 cases) and B (0 case), the rate was not significantly different (*p* = 0.507). During the operation, GTMS was not used in 117 (56.8%) patients (Group A) but was used in 89 (43.2%) patients (Group B). PSEH was noted in 31 patients (26.5%) in Group A and 12 patients (13.5%) in Group B, showing a significant difference between the groups (*p* = 0.023). Furthermore, regarding the grading of PSEH, Group A consisted of 86 patients (73.5%) with Grade 0, 10 patients (8.5%) with Grade I, 16 patients (13.7%) with Grade II, and 3 patients (4.3%) with Grade III, while Group B consisted of 77 patients (86.5%) with Grade 0, 9 patients (10.1%) with Grade I, 3 patients (3.4%) with Grade II, and 5 patients (2.4%) with Grade III (Table 2). The VAS-leg score in Group A improved from 7.3 ± 0.7 preoperatively to 3.1 ± 0.85 at 3 months postoperatively and 1.5 ± 1.0 at the final follow-up. The VAS-leg score in Group B improved from 7.4 ± 0.6 preoperatively to 2.0 ± 1.0 at 3 months postoperatively and 1.1 ± 0.9 at the final follow-up. The VAS-leg improvement was significantly different depending on the presence of PSEH. Moreover, the VAS-leg improvement was significantly different depending on the intraoperative use of GTMS at both 3 months postoperatively (*p* = 0.00) and the final follow-up (*p* = 0.03). The VAS-back score in Group A improved from 5.2 ± 0.9 preoperatively to 3.0 ± 1.0 at 3 months postoperatively and 1.6 ± 0.9 at the final follow-up. The VAS-back score in Group B improved from 5.0 ± 1.1 preoperatively to 2.7 ± 0.9 at 3 months postoperatively (*p* = 0.016) and 1.4 ± 0.7 at the final follow-up. Although VAS-back improvement was more significant in Group B at 3 months postoperatively, no significant difference was found at the final follow-up (*p* > 0.05) (Figure 4).

The ODI score in Group A improved from 60.3 ± 6.9 preoperatively to 26.7 ± 8.5 at 3 months postoperatively and 16.0 ± 8.5 at the final follow-up. The ODI score in Group B improved from 60.3 ± 7.4 preoperatively to 18.3 ± 7.2 at 3 months postoperatively and 13.6 ± 6.4 at the final follow-up. The difference in ODI score between the two groups was statistically significant at both 3 months postoperatively (*p* = 0.00) and the final follow-up (*p* = 0.019). According to the modified MacNab criteria, 88 patients (76.1%) responded “excellent” or “good” in Group A, while 78 patients (87.6%) responded “excellent” or “good” in Group B. Group B showed a significantly higher rate of “excellent” or “good” according to modified MacNab criteria (*p* = 0.049) (Figure 3).

## 4. Discussion

**Figure 5:**
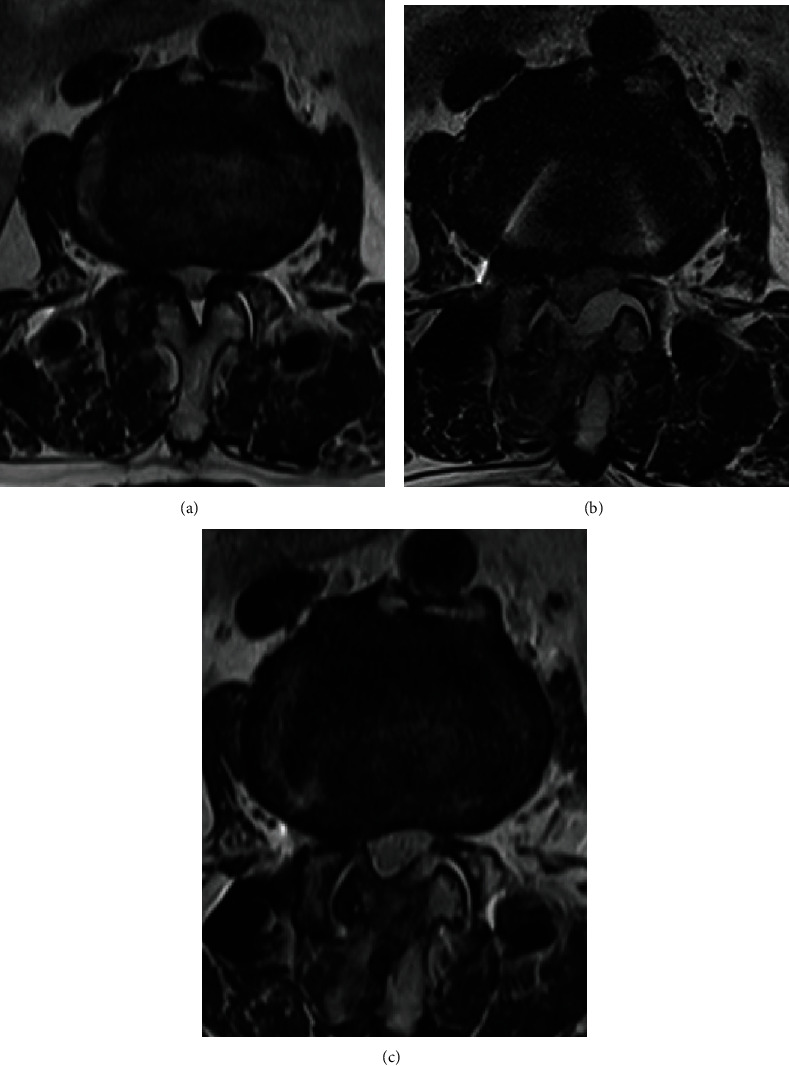
A patient who underwent BESS decompression for L2–3 spinal stenosis (a) Preoperative T2-weighted MRI showing bilateral recess stenosis. (b) Postoperative T2-weighted MRI showing grade II PSEH. (c) Follow-up MRI 6 months after surgery showing resorption of the PSEH with epidural fibrosis causing right lateral recess restenosis. (BESS: biportal endoscopic spine surgery, MRI: magnetic resonance image, PSEH: postoperative spinal epidural hematoma).

PSEH is one of the most critical factors that can cause compression of the spinal canal after lumbar decompression surgery. The incidence of PSEH after lumbar spine surgery detected by MRI and CT is relatively high [16]. However, a significant discrepancy exists between radiological incidence and symptoms of PSEH [15]. PSEH with neural encroachment of at least 50% based on postoperative MRI caused neural injury secondary to decompression and ischemia [14, 15]. Therefore, bleeding control is one of the factors affecting successful clinical outcomes after spine surgery. Owing to recent developments in the techniques of minimally invasive spine surgery and advancements in endoscope-related optical technology, BESS was introduced, and numerous results have been reported [8–13]. Because it has surgical indications similar to those in conventional open surgery, BESS is gaining interest recently as a minimally invasive spine surgery technique with favorable outcomes similar to those of microscopic decompression in spinal stenosis [8]. However, even in BESS, postoperative complications, such as dural tear and PSEH, are being reported [9]. Although symptomatic PSEH is rare, cases of Grade III or higher were reported to cause motor weakness, severe radiating pain, and cauda equina syndrome.^14,15^ Several studies revealed that, to prevent neurological sequelae, removal of symptomatic PSEH is necessary [3]. Although the majority of PSEH resolves in a length of time postoperatively, in some cases, low signal intensity bands are found around the dural sac on MRI. Although decompression was sufficient, as the PSEH is reabsorbed, it can form fibrosis in the epidural space and inhibit dural sac expansion. Epidural fibrosis due to PSEH interferes with permanent dura expansion, which leads to nerve root irritation and worsens neurological symptoms [15]. For these reasons, previous studies reported that the clinical outcome of patients with PSEH was inferior to that of patients without PSEH (Figure 5). Therefore, it is assumed that meticulous hemostasis is directly related to positive clinical outcomes.

Several studies have been conducted to prevent PSEH from developing in open surgery, but no accurate consensus has been reached regarding risk factors and incidence [3]. Similarly, while studies have been conducted on the risk factors of PSEH in BESS [14], a practical prevention method has not been introduced. GTMS is also used when electric coagulation does not achieve adequate hemostasis. Passive hemostatic agents, such as collagen and gelatin, induce contact activation and platelet aggregation to stop bleeding, while active hemostatic agents, such as fibrin sealant, act biologically on clotting mechanisms [7, 17]. To achieve adequate hemostasis, forming a stable clot consisting of two independent hemostatic agents, bovine-derived gelatin granule and human thrombin, is useful [17].

In this study, the incidence of PSEH and clinical outcomes were investigated by comparing the group in which GTMS was used and the group in which GTMS was not used during single-level decompression in spinal stenosis. In the group in which GTMS was used, the incidence of PSEH was decreased by half compared to the group in which GTMS was not used, and there was no revision surgery due to PSEH. Additionally, while the overall rate of PSEH was >20%, high-grade PSEH (Grade III or IV) was noted in 5 patients in Group A and 0 case in Group B. The two patients in Group A with high-grade PSEH underwent revision surgery. This result supports the use of GTMS in preventing high-grade PSEH. Although the follow-up period was short, significant differences in the clinical outcomes were observed. If PSEH is present, it causes not only dural compression but also histopathological changes, such as inflammation, adhesion, and fibrosis, which can lead to nerve traction and arterial supply impairment, resulting in radiculopathy [18]. Epidural fibrosis can cause back or lower extremity pain up to 6 months postoperatively [19].

Other study results regarding epidural fibrosis after the use of hemostatic agents vary. One study reported that a granule-type GTMS can cause histopathological changes, such as inflammation, when left near the dura [20]. In contrast, an experiment on rat models showed that epidermal fibrosis did not significantly differ between the groups in which polysaccharides were used and not used during laminectomy, concluding that an absorbable polysaccharide hemostatic agent did not promote epidural fibrosis formation [21]. In another animal experiment, the histopathological examination showed less epidural fibrosis in the group in which GTMS was not used [22].

In our case series, at the end of the surgery, integrated hematoma and granule were removed using continuous saline irrigation 3 min after administration of GTMS on the spinal canal. As previously mentioned, a postoperative residual granule from GTMS is recognized as a foreign body that may cause inflammation and granuloma to form, resulting in radicular pain.^20^ Thus, a recent study recommends flowable hemostatic agents, such as gelatin granule and thrombin, rather than granule-type agents [20]. The strength of our study is that, for the first time, the effect of GTMS use in preventing PSEH during BESS was evaluated and found to decrease the incidence by half. It was also noted that the presence of PSEH was negatively related to clinical outcomes. Proper use of flowable hemostatic agents, such as GTMS, can effectively stop bleeding without adverse effects, in not only open spine surgery but also endoscopic spine surgery. However, there are limitations in this study. First, a sampling bias was considered because cases performed in the early period of BESS were included in Group A, which might have poorer outcomes compared to the latter cases, Group B, based on the learning curve. Nonetheless, all cases in this study can be considered adequately performed since they were performed by a single surgeon with experience of at least 60 cases [23]. Second, performance comparison of different hemostatic products was not possible because only a single product GTMS was used in this study.

## 5. Conclusions

Intraoperative use of GTMS during BESS may be related to a reduction in the occurrence rate of PSEH. Specifically, patients with GTMS appliance showed marked decrease in the occurrence of PSEH based on postoperative MRI and had better clinical outcomes, such as VAS-leg and ODI scores and modified MacNab criteria.

## Figures and Tables

**Table 1:** Clinical and demographic data of patients.

	Group A (*n* = 117)	Group B (*n* = 89)
Age		
Mean (year)	67.9 ± 10.6	67.6 ± 11.7
Range (year)	36~86	23~71
Gender		
Male	62 (53.0%)	37 (41.6%)
Female	55 (47.0%)	52 (58.4%)
Operate level		
L2~L3	2 (1.7%)	2 (2.2%)
L3~L4	13 (11.1%)	15 (16.9%)
L4~L5	96 (82.1%)	67 (75.3%)
L5~S1	6 (5.1%)	5 (5.6%)

**Table 2:** Incidence of PSEH after BESS by grading system and the incidence of revision surgery caused by PSEH. (PSEH: postoperative spinal epidural hematoma, BESS: biportal endoscopic spine surgery).

	Total patients *n* = 206	Group A *n* = 117 (56.8%)	Group B *n* = 89 (43.2%)
Non-PSEH (Grade 0)	169 patients (79.1%)	86 patients (73.5%)	77 patients (86.5%)
PSEH (Grades I~IV)	43 patients (21.9%)		
Grade I (canal encroachment 0~25%)	19 patients (9.2%)	10 patients (8.5%)	9 patients (10.1%)
Grade II (canal encroachment 50~75%)	19 patients (9.2%)	16 patients (13.7%)	3 patients (3.4%)
Grade III (canal encroachment 50~75%)	5 patients (2.4%)	5 patients (4.3%)	0 patients (0.0%)
Grade IV (canal encroachment 50~75%)	0 patients (0%)	0 patients (0%)	0 patients (0.0%)
Revision due to PSEH	2 patients (0.9%)	2 patients (1.7%)	0 patients (0.0%)

## Data Availability

All equipment and materials used in this work are described, and all relevant results obtained were presented and discussed (tables). Other details can be requested.

## References

[1] Aono H., Ohwada T., Hosono N. (2011). Incidence of postoperative symptomatic epidural hematoma in spinal decompression surgery. *Journal of Neurosurgery. Spine*.

[2] Yamada K., Abe Y., Satoh S., Yanagibashi Y., Hyakumachi T., Masuda T. (2015). Large increase in blood pressure after extubation and high body mass index elevate the risk of spinal epidural hematoma after spinal surgery. *Spine*.

[3] Amiri A. R., Fouyas I. P., Cro S., Casey A. T. H. (2013). Postoperative spinal epidural hematoma (SEH): incidence, risk factors, onset, and management. *The Spine Journal*.

[4] Ma L., Dai L., Yang Y., Liu H. (2018). *Medicine (Baltimore)*.

[5] Skaf G., Bouclaous C., Alaraj A., Chamoun R. (2005). Clinical outcome of surgical treatment of failed back surgery syndrome. *Surgical Neurology*.

[6] Gazzeri R., Galarza M., Neroni M., Alfieri A., Giordano M. (2011). Hemostatic matrix sealant in neurosurgery: a clinical and imaging study. *Acta Neurochirurgica*.

[7] Echave M., Oyagüez I., Casado M. A. (2014). Use of Floseal®, a human gelatine-thrombin matrix sealant, in surgery: a systematic review. *BMC Surgery*.

[8] Min W.-K., Kim J.-E., Choi D.-J., Park E. J., Heo J. (2020). Clinical and radiological outcomes between biportal endoscopic decompression and microscopic decompression in lumbar spinal stenosis. *Journal of Orthopaedic Science*.

[9] Kim J. E., Choi D. J., Park E. J. J. (2019). Biportal endoscopic spinal surgery for lumbar spinal stenosis. *Asian Spine J.*.

[10] Yamauchi T., Kim K., Isu T. (2018). Undiagnosed peripheral nerve disease in patients with failed lumbar disc surgery. *Asian Spine Journal*.

[11] Kim J. E., Choi D. J., Park E. J. (2018). Clinical and radiological outcomes of foraminal decompression using unilateral biportal endoscopic spine surgery for lumbar foraminal stenosis. *Clinics in Orthopedic Surgery*.

[12] Kim J. E., Choi D. J. (2018). Clinical and radiological outcomes of unilateral biportal endoscopic decompression by 30° arthroscopy in lumbar spinal stenosis: minimum 2-year follow-up. *Clinics in Orthopedic Surgery*.

[13] Kim J. E., Choi D. J. (2018). Biportal endoscopic transforaminal lumbar interbody fusion with arthroscopy. *Clinics in Orthopedic Surgery*.

[14] Kim J. E., Choi D. J., Kim M. C., Park E. J. (2019). Risk factors of postoperative spinal epidural hematoma after biportal endoscopic spinal surgery. *World Neurosurgery*.

[15] Kim J. E., Choi D. J., Park E. J. (2019). Evaluation of postoperative spinal epidural hematoma after biportal endoscopic spine surgery for single-level lumbar spinal stenosis: clinical and magnetic resonance imaging study. *World Neurosurgery*.

[16] Ikuta K., Tono O., Tanaka T. (2006). Evaluation of postoperative spinal epidural hematoma after microendoscopic posterior decompression for lumbar spinal stenosis: a clinical and magnetic resonance imaging study. *Journal of Neurosurgery:Spine*.

[17] Yao H. H. I., Hong M. K. H., Drummond K. J. (2013). Haemostasis in neurosurgery: what is the evidence for gelatin-thrombin matrix sealant?. *Journal of Clinical Neuroscience*.

[18] Nishida K., Kakutani K., Maeno K. (2013). Efficacy of hemostasis for epidural venous plexus and safety for neural structure using soft coagulation system in spinal surgery: a laboratory investigation using a porcine model. *Journal of Spinal Disorders & Techniques*.

[19] Zhang C., Kong X., Zhou H. (2013). An Experimental Novel Study: Angelica sinensis Prevents Epidural Fibrosis in Laminectomy Rats via Downregulation of Hydroxyproline, IL-6, and TGF-*β*1. *Evidence-based Complementary and Alternative Medicine*.

[20] Altun I. (2016). An experimental study of histopathologic effects of hemostatic agents used in spinal surgery. *World Neurosurgery*.

[21] Tural Emon S., Somay H., Orakdogen M., Uslu S., Somay A. (2016). Effects of hemostatic polysaccharide agent on epidural fibrosis formation after lumbar laminectomy in rats. *The Spine Journal*.

[22] Gurcan O., Gurcay A. G., Kazanci A., Onder E., Senturk S., Bavbek M. (2017). Is the use of hemostatic matrix (Floseal) and alkylene oxide copolymer (Ostene) safe in spinal laminectomies? Peridural fibrosis assessment. *Acta Orthopaedica et Traumatologica Turcica*.

[23] Park S.-M., Kim H.-J., Kim G.-U. (2019). Learning curve for lumbar decompressive laminectomy in biportal endoscopic spinal surgery using the cumulative summation test for learning curve. *World Neurosurgery*.

